# Programmable Millifluidic Platform Integrating Automatic Electromembrane Extraction Cleanup and In-Line Electrochemical Detection: A Proof of Concept

**DOI:** 10.1021/acssensors.2c01648

**Published:** 2022-10-06

**Authors:** Ali Sahragard, Miloš Dvořák, Enrique J. Carrasco-Correa, Pakorn Varanasupakul, Pavel Kubáň, Manuel Miró

**Affiliations:** † Department of Chemistry, Faculty of Science, Chulalongkorn University, Bangkok10330, Thailand; ‡ Institute of Analytical Chemistry of the Czech Academy of Sciences, Veveří 97, BrnoCZ-60200, Czech Republic; § CLECEM group, Department of Analytical Chemistry, University of Valencia, C/Doctor Moliner 50, Burjassot, Valencia46100, Spain; ∥ FI-TRACE Group, Department of Chemistry, Faculty of Science, University of the Balearic Islands, Carretera de Valldemossa km 7.5, Palma de Mallorca, Illes BalearsE-07122, Spain

**Keywords:** electrochemical sensing, nonsupported electrically driven extraction, diclofenac, automation, sequential injection analysis

## Abstract

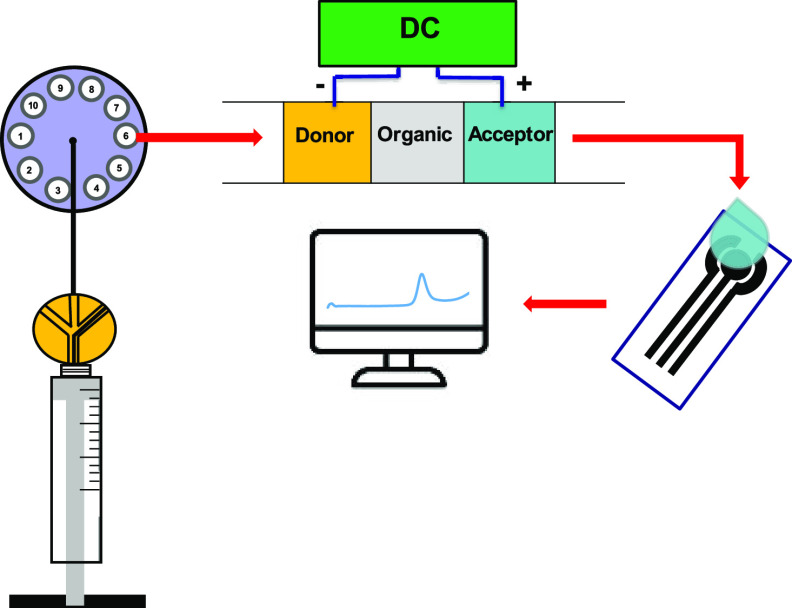

A fully automatic millifluidic sensing platform coupling in-line nonsupported microelectromembrane extraction (μ-EME) with electrochemical detection (ECD) is herein proposed for the first time. Exploiting the features of the second generation of flow analysis, termed sequential injection (SI), the smart integration of SI and μ-EME–ECD enables (i) the repeatable formation of microvolumes of phases for the extraction step in a membrane-less (nonsupported) arrangement, (ii) diverting the acceptor plug to the ECD sensing device, (iii) in-line pH adjustment before the detection step, and (iv) washing of the platform for efficient removal of remnants of wetting film solvent, all entirely unsupervised. The real-life applicability of the miniaturized sensing system is studied for in-line sample cleanup and ECD of diclofenac as a model analyte after μ-EME of urine as a complex biological sample. A comprehensive study of the merits and the limitations of μ-EME solvents on ECD is presented. Under the optimal experimental conditions using 14 μL of unprocessed urine as the donor, 14 μL of 1-nonanol as the organic phase, and 14 μL of 25 mM NaOH as the acceptor in a 2.4 mm ID PTFE tubing, an extraction voltage of 250 V, and an extraction time of 10 min, an absolute (mass) extraction recovery of 48% of diclofenac in urine is obtained. The proposed flow-through system is proven to efficiently remove the interfering effect of predominantly occurring organic species in human urine on ECD with RSD% less than 8.6%.

A broad range of liquid-phase and sorptive (micro)extraction approaches encompassing solid phase extraction (SPE), solid phase microextraction (SPME), and liquid–liquid extraction (LLE) has been developed over the last few decades and successfully applied to the cleanup and preconcentration of a plethora of analyte classes.^1^ Notwithstanding the acceptance of the above sample preparation methods in routine analysis and research settings, practitioners still need to cope with the (i) long synthesis protocols of customized sorbents in SPE, (ii) high expenses of commercial microfibers in SPME, and (iii) elevated consumption and waste generation of organic solvents in LLE methods.^2^ To mitigate the lack of green credentials of LLE, dispersive liquid-phase microextraction (DLPME) and supported liquid membrane (SLM)-based LPME methods, such as hollow fiber LPME (HF-LPME) and electromembrane extraction (EME), have been developed as viable alternatives for the extraction of analytes of a broad range of polarity in troublesome biological and environmental samples.^3−7^ EME is a variant of HF-LPME in which charged analytes are extracted from sample solutions based on their electrically driven migration through the SLM into an acceptor solution. Due to the application of a given voltage as a driving force, EME can generally provide higher enrichment factors in shorter extraction times as compared with conventional HF-LPME of neutral species.^5,8,9^

To address the main challenges for automation of SLM-based methods, such as (i) the limited reusability of SLMs for repetitive experiments,^10,11^ (ii) the frequent leakage of solvents from SLMs,^12^ and (iii) the need for manual impregnation of the SLMs,^13−15^ flow injection methods and variants thereof (e.g., sequential injection analysis (SI)) have been assembled for handling the solutions unattended^16−18^ including the regeneration of the SLM or the usage of a new plug of organic membrane in every single run.^19,20^ However, coupling with bulk instrumentation, such as chromatographic equipment, which does not enable decentralized assays, is often reported.^19,20^ To tackle this limitation, electrochemical detection (ECD) can be seen as a fast, flexible, and sensitive sensing alternative for portable setups. Because of the similar redox potentials of electroactive organic compounds, samples with complicated matrices can however have deleterious effects on the electrochemical readouts. To this end, several off-line LPME approaches, e.g., DLPME,^21−23^ HF-LPME,^24−26^ and EME,^27−30^ have been combined with ECD, including smart designs for *in situ* microextraction and detection of various analytes.^26,29−31^ On the other hand, there has been no attempt at automation in terms of sample handling based on LPME in combination with ECD, only a membrane-based platform exploiting centrifugal microfluidics.^32^ Further, to the best of our knowledge, there is no report in the literature leveraging EME as a “front end” to ECD. The lack of LPME/EME-ECD couplings using flow systems is probably a consequence of the susceptibility of the electrochemical signals to deteriorate in the presence of even trace amounts of organic solvents used as liquid membranes or cleaning organic agents for removal of solvent remnants (e.g., wetting films on the polytetrafluoroethylene (PTFE) walls).

In this work, an automatic flow system based on the coupling of SI and nonsupported μ-EME (downscaled version of EME that copes with green chemical principles and is amenable to automation) with electrochemical detection (SI−μ-EME–ECD) is proposed for the first time. Nonsupported μ-EME (also called μ-EME through free liquid membrane) consists of a plug of an organic solvent, which is inserted between a plug of donor and acceptor solution with no need for permeable membranes.^33,34^ The analytical millifluidic platform capitalizes upon programmable flow using a user-friendly software for (i) in-line unattended handling of the overall solvent/sample plugs in the μ-EME unit and formation of the three phases reliably, (ii) retrieval and analyzing of the analyte-containing acceptor phase, and (iii) automatic manipulation of the buffer solution for *in situ* pH adjustment in the ECD cell prior to sensing. The SI−μ-EME–ECD hyphenation was applied to the determination of diclofenac as a model analyte in urine samples on the basis of which the key parameters of the μ-EME approach influencing the SI network and the ECD performance were studied in detail.

## Experimental Section

### Reagents, Standard Solutions, and Real Samples

The information about this part is provided in the Supporting Information.

### Flow Setup for Automatic μ-EME and ECD

**Figure 1:**
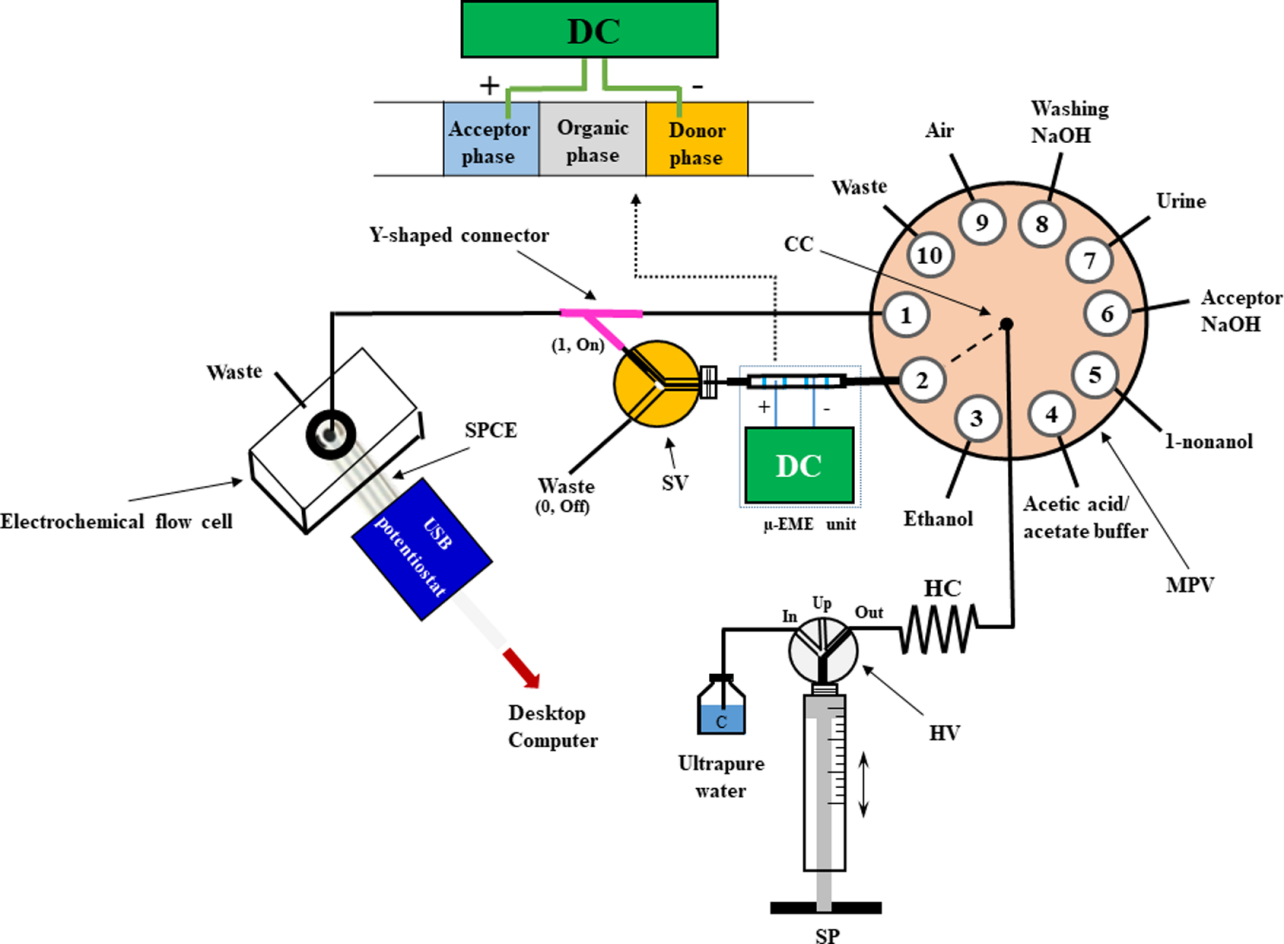
Diagrammatic description of the SI – μ-EME – ECD hyphenation. SP, syringe pump; MPV, multiposition valve; HV, head valve; HC, holding coil; CC, central channel; μ-EME, microelectromembrane extraction; DC, direct current power supply; SPCE, screen printed carbon electrode; SV, solenoid valve.

A diagrammatic description of the flow manifold incorporating μ-EME and flow-through ECD is shown in Figure 1 and the close-up of the assembled SI−μ-EME–ECD system is provided in Figure S1. The millifluidic SI-based device (microSIA, FIAlab Instruments, Seattle, WA, USA) is composed of a 10-port multi-position selection valve (MPV) and a 30 mm-stroke bidirectional microsyringe pump (SP) with a 24 V output for peripheral device connection, in our case, a three-way solenoid valve (SV, Valcor Scientific, Springfield, NJ, USA). A three-port (In, Up, and Out) head valve (HV) enabled SP to aspirate the carrier solution (Milli-Q, In position) and air (Up position) and to communicate with the flow manifold via a 19 cm-long holding coil (HC, 1.0 mm ID, 1.6 mm OD PTFE tubing, Idex Health and Science LLC, Oak Harbor, WA, USA, connected to the Out position). A 250 μL-borosilicate glass syringe (Cavro Scientific Instruments, San Jose, CA, USA) was used for automatic aspiration and pumping of the overall solutions and air. Computer-controlled and programmable aspiration of the samples/standards, acceptor phase, cleaning solvent, and renewable organic phase was carried out using external ports (1–10, see Figure 1) through the MPV central channel (CC) into HC. A 2 cm-long PTFE tubing (2.4 mm ID, 3.2 mm OD, Idex Health and Science LLC, port #2), employed for in-line μ-EME experiments, was connected to the MPV (port #2) by a 2 cm transfer line (1.6 mm ID, 2.4 mm OD PTFE tubing, Idex Health and Science LLC). To connect the μ-EME unit to the SV, a 0.5 cm of 1.6 mm ID PTFE tubing (2.4 mm OD PTFE, Idex Health and Science LLC) was first inserted into the μ-EME tubing. The other end of the 1.6 mm ID tubing communicated to the SV via a 2.8 cm transfer line (0.76 mm ID, 1.6 mm OD PTFE tubing, Idex Health and Science LLC). Position 1 (On) of the SV was connected to a Y-shaped connector, which served for directing a given volume of acceptor phase and the acetate buffer stream (port #1) toward the ECD. The output of the Y-shaped connector was connected to the electrochemical flow cell (Metrohm DropSens, Oviedo, Spain) by a 3 cm PTFE transfer line (1.6 mm ID, 2.4 mm OD PTFE tubing, Idex Health and Science LLC). The electrochemical flow cell is composed of an inlet channel with a length of 1.5 cm and an ID of 1.5 mm and a parallel outlet channel with a length of 1.0 cm and an ID of 1.5 mm.

The open-source software Cocosoft (version CS71) was used to unite all parts of the flow system including SP, MPV, and SV as well as to control the μ-EME power supply (DC) and the electrochemical software (PStrace).^35^

### Electrochemical Detection

A USB potentiostat/galvanostat (EmStat^3+^, PalmSens, Houten, The Netherlands) with the associated PStrace 5.9 software (PalmSens) was used for all electrochemical measurements and data processing. Screen-printed carbon sensors/electrodes (I-SC code) were also purchased from PalmSens. Each sensor consisted of a working and a counter electrode, both made of carbon, and a pseudo-reference electrode based on silver. A 2 mm banana (PalmSens) was used for connection of the screen-printed carbon electrodes (SPCE) to the potentiostat/galvanostat. A methacrylate-based commercial electrochemical flow-through cell (Metrohm DropSens) for screen-printed electrodes was used for electrochemical sensing. As can be seen in Figure 1 and Figure S1, a round flexible black rubber washer (ID of 7 mm and OD of 10 mm, 3G Hidraulica, Palma, Spain) was placed onto the SPCE for confining the three electrodes, which were sandwiched between the washer and the bottom part of the flow cell. The lag screws were tightened to an extent that ensures a total volume of 18 μL in the electrochemical flow cell (the thickness of the washer was set to 0.64 mm (distance between the electrode and the upper part of the methacrylate cell) and the inner diameter to *ca.* 6 mm after screwing). In order to replace the spent electrodes with fresh electrodes, the electrochemical flow cell was unscrewed and the same procedure was followed. Voltammetric sensing was performed using 18 μL of the final solution containing *ca.* 9 μL of acceptor phase and *ca.* 9 μL of the acetic acid/acetate buffer at pH 3.75. Experimental conditions for the differential pulse voltammetry (DPV) were as follows: voltage range of 0–1 V, scan rate of 100 mV s^–1^, step potential of 10 mV, pulse amplitude of 50 mV, pulse time of 10 ms with −0.25 V and 60 s as the accumulation potential and time, respectively (see voltammogram example in Figure S2 in the Supporting Information).^36^ DPV curves were baseline normalized by the PStrace software for the sake of quantitative data processing. Normalized curves were obtained by the ratio of the voltammogram currents to the estimated baseline currents at each voltage. In-line washing with the acetic acid/acetate buffer at pH 3.75 (95 μL) was used for electrode conditioning and regeneration.

**Figure 2:**
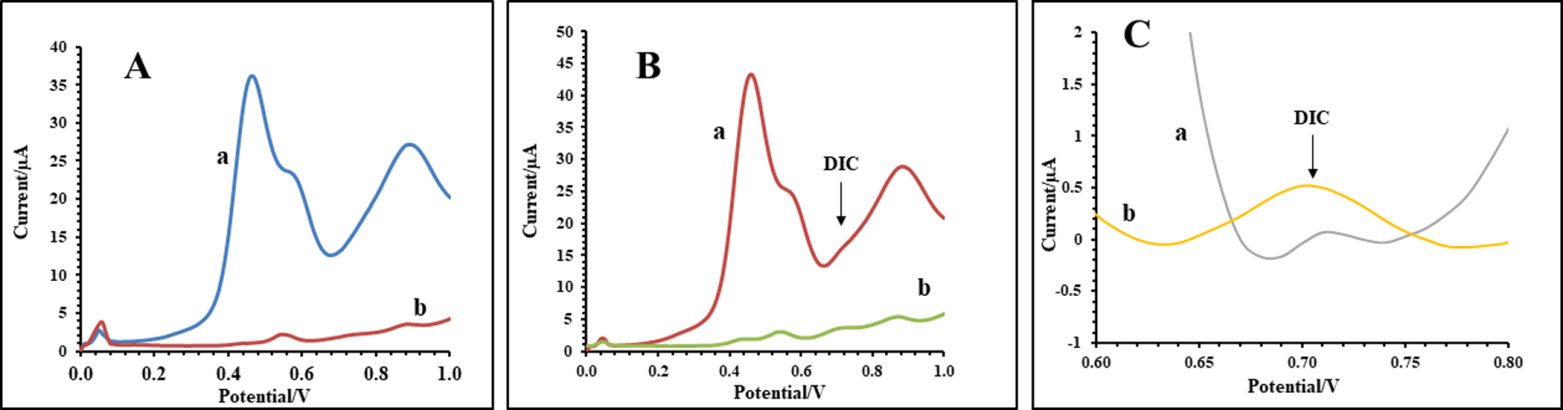
**(**A) DPV curves of (a) direct sensing of blank urine, (b) sensing blank urine after SI – μ-EME, (B) DPV curves of (a) direct sensing of unprocessed urine spiked with 2.5 mg L^–1^ of diclofenac, (b) sensing urine spiked with 2.5 mg L^–1^ of diclofenac after SI – μ-EME, and (C) magnified view of the baseline normalized DPV curves of (a) direct sensing of unprocessed urine spiked with 2.5 mg L^–1^ of diclofenac, (b) urine spiked with 2.5 mg L^–1^ of diclofenac after SI – μ-EME. Extraction conditions: acceptor solution, 14 μL of 25 mM NaOH; organic solvent; 14 μL of 1-nonanol; donor solution, 14 μL of urine sample; extraction voltage, 250 V; and extraction time, 10 min.

**Figure 3:**
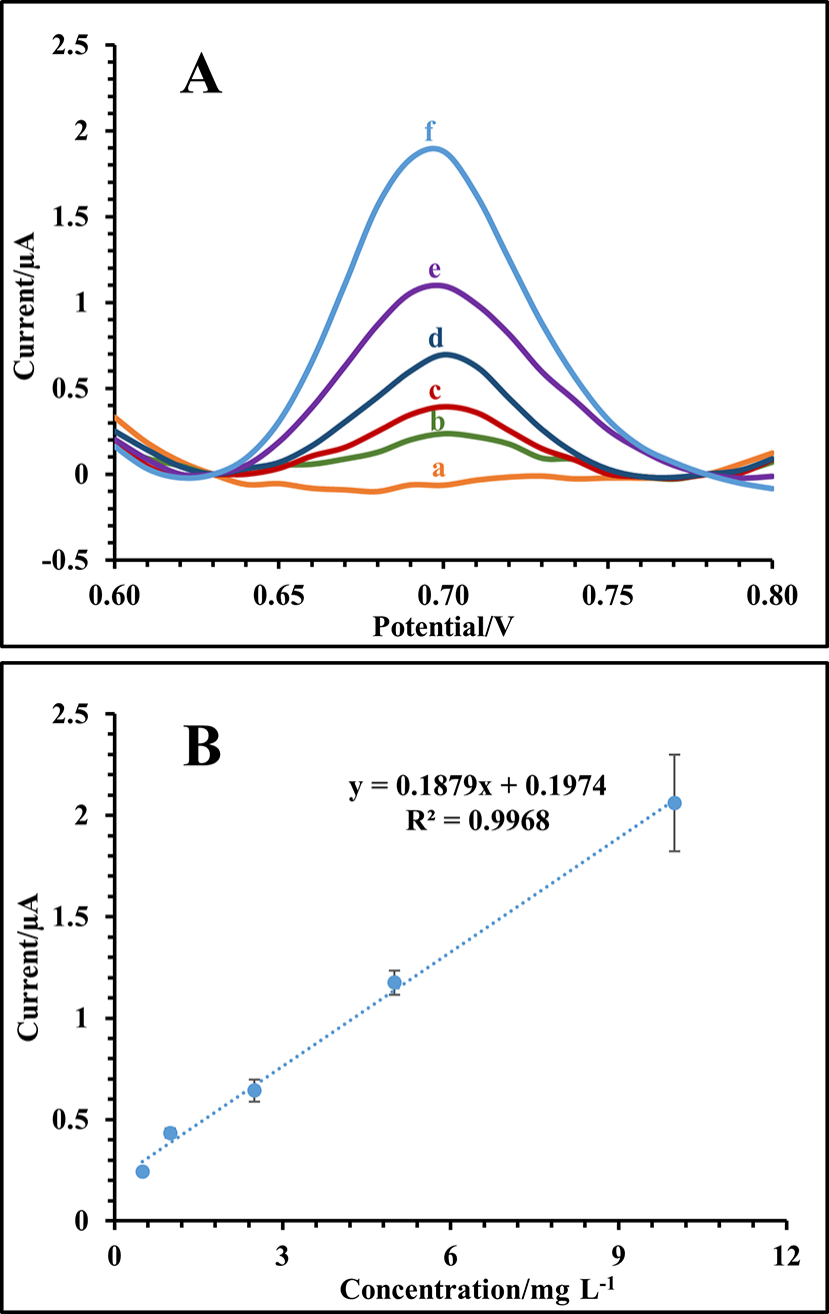
(A) Baseline normalized DPV curves of SI – μ-EME–ECD of diclofenac concentrations in urine on SPCE ((a) 0, (b) 0.5, (c) 1.0, (d) 2.5, (e) 5, and (f) 10 mg L^–1^) and (B) linear range. Extraction conditions: donor solution, 14 μL of unprocessed urine sample containing various diclofenac concentrations; acceptor solution, 14 μL of 25 mM NaOH; extraction solvent, 14 μL of 1-nonanol; extraction voltage, 250 V; extraction time, 10 min.

### In-Line μ-EME

Automatic nonsupported μ-EMEs were performed in chemically inert PTFE tubing (2.4 mm ID and 3.2 mm OD). Two 5 mm long and 500 μm-thick tubular platinum wires (99.95%, Advent RM, Oxford, UK) acted as μ-EME electrodes. These electrodes were fixed as follows: First, a hypodermic needle (0.45 mm OD, Braun, Melsungen, Germany) was used to pierce two holes at a distance of 7 mm from each other on the top of the PTFE tubing (protruded by *ca.* 200 μm into the tubing), and then electrodes were inserted and fixed using a drop of a photopolymerizable resin followed by UV polymerization (Figure 1 and Figure S1) (clear resin, Form 3, Formlabs, Sommerville, MA, USA).

The aqueous donor, organic phase, and aqueous acceptor solutions were automatically provided by the SI system so that the anode and the cathode were in all instances in contact with the acceptor and the donor solutions, respectively. The programmable voltage for μ-EME within the range of 0–300 V was provided by a DC power supply, ES 0300–0.45 (Delta Elektronika, Zierikzee, The Netherlands) that incorporated a printed circuit board (version P148) for RS232 communication. Electric currents in the time course of the μ-EME were monitored using a UT70B (Uni-Trend Technology Ltd., Dongguan, China) digital multimeter.

### Analytical Operational Procedure for μ-EME-ECD

The hyphenated analytical method capitalizing on programmable SI involves the following steps, and the detailed sequence is provided in Table S1 in the Supporting Information:

(i) Automatic formation of the three μ-EME phases: A flow of air (2 × 250 μL) from MPV (port #9 at 600 μL min^–1^) was used to empty the μ-EME PTFE tubing and the transfer line toward the detection system. Then, a step-by-step methodology was implemented to introduce the three μ-EME phases into the μ-EME tubing. First, SP and MPV were programmed to aspirate consecutively 40 μL of air (port #9) and 14 μL of acceptor solution (port #6) into the HC at 500 μL min^–1^ (HV was set to Out). To avoid mixing of the next aspirated solutions into the HC with the carrier, the air segment remained in the HC, while the acceptor phase was dispensed at 300 μL min^–1^ into the μ-EME tubing (port #2). An identical protocol was performed for the organic phase (14 μL, port #5, 30 μL min^–1^) and the urine sample (14 μL, port #7, 30 μL min^–1^). As the organic solvent could be easily scattered onto the wall of tubes at high flow rates with the consequent unwanted formation of an organic wetting film, handling the organic phase was always carried out at a flow rate of 30 μL min^–1^ in all steps. Needless to say, this was also applied to all solutions handled across port #2 including donor solution, air, ethanol (washing solution), and carrier in the μ-EME step. After the urine segment was dispensed into the μ-EME tubing, 60 μL of 25 mM NaOH (port #8, at 500 μL min^–1^) and 250 μL of Milli-Q water (1500 μL min^–1^, aspirated from In position) were consecutively aspirated and dispensed to waste (port #10) to assure removal of the urine leftovers from HC.
(ii) Execution of μ-EME: A sequential protocol is programmed for the aspiration and dispensing into the μ-EME tubing of 35 μL of air (port #9, 30 μL min^–1^), and 50 μL of ethanol (port #3, 30 μL min^–1^) so that the three μ-EME plugs are followed by a segment of washing solvent separated by an air plug. Then, all segments were dispensed toward the electrodes, and the flow was stopped whenever the organic phase was placed in between the cathode and the anode, whereupon the DC was automatically activated at 250 V for 10 min.
(iii) In-line injection of the acceptor solution into the electrochemical flow cell and detection: After μ-EME, the DC was automatically switched off, and the SP was programmed to dispense all the plugs forward toward the sensing system via the SV aiming at isolating *ca.* 9 μL of the analyte-containing acceptor phase while flushing the rest of the acceptor phase, the organic phase, and the donor phase to the waste by the ensuing air and ethanol plugs. It is necessary to explain here that the incorporation of SV was essential for the efficient retrieval of the acceptor phase and removal of the other two phases, especially the organic solvent, otherwise, passing the organic phase through the electrochemical flow cell would compromise the reusability of the electrodes. After bringing the unwanted solutions to waste, the acceptor plug is moved to the electrochemical flow cell by air at a flow rate of 250 μL min^–1^. In the electrochemical flow cell, the alkaline acceptor phase is mixed with the pre-existing buffer solution introduced after each complete washing of the electrochemical flow cell described in the following: first, 100 μL of 25 mM NaOH and then 500 μL of water are passed through the electrochemical flow cell to eliminate the potential remnants of acceptor phase from the previous run, followed by their flush to waste with 500 μL of air. Afterward, 95 μL of a buffer is passed through the electrochemical cell and then flushed out by air (240 μL). The remaining *ca.* 9 μL buffer in the electrochemical flow cell after air flush is used for pH adjustment (the final pH is ∼4).
(iv) To fully synchronize the automatic flow-through μ-EME and ECD, at the time that the retrieved acceptor solution arrives at the electrochemical cell, the Cocosoft software can activate the PStrace software exploiting the so-called *Click ()* function^35^ so that the DPV signals are recorded and autosaved in the computer. The main advantage of this synchronization protocol is that both instruments operate simultaneously with their own software, and thus the sample throughput can be maximized.

## Results and Discussion

### Sample Cleanup

To evaluate the relevance of implementing a urine cleanup step prior to the ECD, the readouts of direct sensing of unprocessed urine (blank) and urine containing 2.5 mg L^–1^ diclofenac were compared with those obtained through SI−μ-EME–ECD. As can be seen in Figure 2A-a,B-a, high amounts of organic species in urine such as ascorbic acid, dopamine, and uric acid covered a wide range of the electrochemical window in ECD and the signal of diclofenac was not quantifiable (Figure 2B-a).^37,38^ However, after μ-EME, the diclofenac peak appears clearly at ∼0.7 V without being influenced by neighboring signals (Figure 2B-b). Figure 2C illustrates a magnified view of the baseline normalized signal of diclofenac as obtained by direct sensing of urine against that after μ-EME. Cleanup of urine interfering electroactive compounds with an electrochemical potential similar to that of diclofenac enables a reliable ECD of diclofenac in urine. Except for a very few reports in the literature,^32^ cleanup methods have not been investigated in the couplings between microextraction studies and ECD. Rather, large dilution factors before or after LPME have been applied before analyzing real samples by ECD to mitigate matrix effects,^31^ which is contradictory to the main purpose of sample preparation using (micro)extraction systems. A comparative study was also carried out herein to evaluate the cleanup efficiency of the SI-μ-EME method. To this end, a blank urine sample and a saline solution containing 100 mM NaCl to mimic the ionic strength of human urine samples^20^ were both subjected to the SI-μ-EME process, and the obtained acceptor phases were then spiked with 10 mg L^–1^ of diclofenac prior to electrochemical sensing. The average DPV peak currents were 7.19 and 7.25 μA for urine and saline solutions, respectively. Therefore, there was almost no difference between peak currents, which suggests a satisfactory cleanup efficiency in urine by the proposed automatic fluidic method.

### Study of Solvent Effect on μ-EME Efficiency and ECD

In order to investigate the effect of different organic solvents on the extraction efficiency of μ-EME of diclofenac, 1-octanol, 1-nonanol, and 1-decanol were assessed using unprocessed urine containing 10 mg L^–1^ diclofenac as the donor sample. Because the solubility of the solvents is quite different in water (and our system leverages aqueous acceptor phases), namely, 0.54 g L^–1^ for 1-octanol, 0.14 g L^–1^ for 1-nonanol, and 0.037 g L^–1^ for 1-decanol at 25 °C,^39^ the ECD backgrounds were expected to be solvent dependent. As shown in Figure S3a, the background signal significantly increased for 1-octanol (from around 0.6 μA up to 120 μA in ECD), and the ECD peak of diclofenac was stifled under the solvent effect. Because of the lack/limited conductivity of 1-octanol, the background and the current levels of dissolved octanol in ECD were supposed to be low, yet increased in practice, which may indicate the generation of oxidized species from 1-octanol during the μ-EME steps under the application of 250 V. In fact, if solvent solubility were the only responsible factor of the observed ECD effects, background readouts should have been also recorded for 1-nonanol on account of its distinct solubility in water compared to 1-decanol, yet this was not the case. 1-Nonanol and 1-decanol provided ECD backgrounds comparable to that of the background buffer. DPV peak currents for 1-nonanol exhibited ∼1.4 times more sensitive response compared with 1-decanol. The extraction recovery (ER%, see formula in SI) obtained by external calibration was 48% for 1-nonanol vs 33% for 1-decanol (Figure S3 inset). These results can be explained by the selectivity of organic solvents, which is alkyl chain length dependent.^33^ The higher the polarity of a particular solvent, the higher is the flux of ionic species. In these experiments, μ-EME currents changed between 3.5 and 8 μA for 1-octanol, 1.5 and 5.5 μA for 1-nonanol, and 0.5 and 2 μA for 1-decanol. Finally, 1-nonanol was selected for subsequent studies.

### Donor, Acceptor, and Organic Phase Volumes

For reliable handling, separation, and displacement of the three nonsupported phases through the manifold tubing while obtaining the highest extraction efficiency, different volumes of the donor and acceptor phases at a ratio of 1:1 were studied from 10 to 14 μL (2.2 to 3.1 mm segment length). However, no significant changes in extraction efficiency (variations down to 2%) were observed, thereby indicating that the mass transfer is directly proportional to the donor volume, within the investigated range, with the electromigration through the organic barrier as the limiting step of the microextraction process. μ-EME through selective nonsupported liquid membranes works best with short plugs (∼2–3 mm) of aqueous solutions,^40^ yet extraction recoveries (ERs) are expected to considerably decrease for donors above 5 mm long. The volume (length) of the organic phase was also studied from 10 to 14 μL in terms of stability of phases through the physical movement of plugs from MPV to the SV and the tolerance of high electric voltages required for a successful μ-EME without phase collapse. Given the observations on repeatability in phase formation with electric currents down to 8 μA for voltages up to 300 V, 14 μL of 1-nonanol could fulfill all of the requirements. Therefore, plugs of 14 μL for all three phases were selected for the subsequent experiments.

### Tubing Materials

The information about this part is provided in the Supporting Information.

### Voltage and Time

With an increase of the applied voltage in μ-EME, extraction recoveries improved up to 250 V and then started to decrease (see Figure S4A in the Supporting Information). Notwithstanding the fact that the electrolysis in the acceptor phase is not expected to decrease the pH significantly (maximum production of H^+^ at 5 μA for 10 min in 14 μL of 25 mM NaOH will be less than 5 mM)^41^ and thus will not jeopardize the unidirectional electromigration of diclofenac, a partial decomposition of the analyte is most likely occurring at about 300 V. The voltage was finally set to 250 V. The extraction time was also studied in the range of 0 to 15 min. Mass recoveries increased from 0 to 48% until 10 min and plateaued afterward (see Figure S4B in the Supporting Information). An extraction time of 10 min was thus selected as optimum for all subsequent experiments.

### pH Control

In order to modify the pH of the alkaline acceptor phase for appropriate in-line electrochemical sensing of the target species, a buffer solution was added to the MPV in port#4 as shown in Figure 1. Herein, in the first strategy, 9 μL of 0.2 M acetic acid/acetate buffer at pH 3.75 was aspirated and dispensed into the electrochemical flow cell through the Y-shaped connection before performing μ-EME extractions. The final pH of the mixture of the buffer solution and 9 μL of the acceptor solution was ∼4 as this is proven the most appropriate for DPV of diclofenac.^36^ However, due to the remaining dead volume in the electrochemical flow cell from the previous washing step, the buffer was diluted and the results were not consistent. To obtain reliable results, as an alternative strategy, the electrochemical flow cell was filled with 95 μL of 0.2 M acetic acid/acetate buffer at the beginning of the analytical protocol. After flushing the buffer with air to the waste, a *ca.* 9 μL buffer was left for mixing with the acceptor solution afterward.

### Investigation of Carry-over Effects

Another vital point for reliable performance of the fluidic sensing system is to rinse the acceptor pathway toward the ECD cell so as to avoid any cross-contamination effect. Our observations signaled that placing the acceptor phase at the front end of the three phases with a forward movement toward ECD enables minimum sample cross-contamination. On the contrary, by applying a forward–backward movement as previously recommended,^20^ with the urine sample either at the front end or rear end of the three plugs, the urine matrix components extracted into the organic wetting film that remained on the PTFE walls contaminated the acceptor phase during the motion of the phases across the μ-EME extraction tube and HC, with the consequent generation of artifact signals in ECD. The forward–backward movement was primarily tested with the aim of fast retrieval of the acceptor phase back to the HC, wherein it could straightforwardly be mixed with minute volumes of buffer and brought to the ECD, thus shortening the total run time by a few minutes.

Removal of the wetting film resulting from the attachment of the organic solvent on the tubing walls was investigated by rinsing the flow system with 50 μL of various organic solvents (acetonitrile, isopropanol, or ethanol) after every individual run. When acetonitrile was used, μ-EME phases collapsed in many experiments, which could be a consequence of the presence of remnants of acetonitrile drops on the walls. Although phases were stable after isopropanol washing, there were still some background signals from urine components in the electrochemical signals. Even after the incorporation of a stopped-flow method to enable sufficient contact time of isopropanol with the PTFE walls for dissolution of the wetting film, artifact signals still occurred (see Figure S5 in the Supporting Information). On the other hand, the use of 50 μL ethanol was proven efficient for quantitative in-line removal of the organic wetting film as demonstrated by a stable baseline without ECD readout shifts (see Figure S5 in the Supporting Information).

Based on the previous results, the following experimental conditions were selected for in-line coupling of SI−μ-EME to ECD: acceptor solution, 14 μL of 25 mM NaOH; organic solvent; 14 μL of 1-nonanol; donor solution, 14 μL of unprocessed urine; extraction voltage, 250 V; and extraction time, 10 min. Under these conditions, the μ-EME stable currents increased from 1.5 to 5.5 μA over 10 min of extraction (see Figure S6 in the Supporting Information).

### Method Validation and Analysis of Real Samples

Under the selected experimental conditions of the SI−μ-EME–ECD method for urine analysis, the following figures of merit were estimated: (i) linear dynamic range from 0.5 to 10 mg L^–1^ in a matrix-match format (see Figure 3A,B), (ii) limit of detection (LOD) of 0.18 mg L^–1^ based on the S/N = 3 criterion, (iii) intraday and interday RSD% values of 5.7% (*n* = 3, 5 mg L^–1^) and 6.1% (*n* = 3, 5 mg L^–1^), respectively, using a new electrode in each measurement, and (iv) mass recovery of 48 ± 3% (*n* = 3, 5 mg L^–1^). The reusability of the SPCEs was studied through the in-line SI−μ-EME–ECD system at the 1 mg L^–1^ level. Results indicated that every single electrode could be re-used up to 5 times with an RSD of 4.8% in ECD currents, whereupon background issues over the entire electrochemical window are observed. In some previous SI configurations, the reusability of the working electrodes was proven impracticable.^42^Figure S6 suggests that regardless of the concentration of diclofenac in urine, μ-EME current profiles are almost similar; thereby, the urine itself serves as an ionic strength buffer of the donor phase.

To evaluate the real-life applicability of the SI−μ-EME–ECD method, three urine samples from volunteers were analyzed (see Reagents, standard solutions, and real samples section in the Supporting Information). The recoveries of spiked samples at expected concentrations in human urine^43^ and those used in previous articles^44−46^ along with RSD% are listed in the Table 1. The relative recovery percentage (RR%) was calculated based on the following equation:
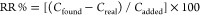
1in which *C*_found_ is the concentration of the analyte detected after sample spiking using a matrix-matched calibration graph performed through μ-EME, *C*_real_ is the concentration of the analyte in the unspiked sample calculated by the matrix-matched calibration, and *C*_added_ is the spike concentration. The relative recoveries obtained ranged from 94 to 106%, thereby corroborating the cleanup efficiency of the automatic system and the lack of significant matrix effects on the ECD. Herein, it should be mentioned that the high level of parent diclofenac existing in the urine sample no. 2 might be due to the syndrome that the urine provider suffers from.

**Table 1:** Automatic Determination of Diclofenac in Urine Samples by SI−μ-EME–ECD after In-Line Cleanup^a^

urine samples	added (mg L^–1^)	found (mg L^–1^)	RSD (*n* = 3) (%)	RR (*n* = 3) (%)
1	0	ND		
	1	0.99	7.2	99.3
	2	1.96	8.6	97.9
2	0	1.64	5.8	
	1	2.65	6.9	101.7
	2	3.53	5.0	94.5
3	0	ND		
	1	1.05	4.5	105.3
	2	1.89	5.4	94.5

a ND, not detected.

Table S2 compares the figures of merit of the SI−μ-EME–ECD method against previous works based on liquid chromatography (LC) or electrochemistry for sensing diclofenac in biological matrixes. Because this study merely serves as a proof of concept of the feasibility of coupling μ-EME with in-line ECD, there was not any effort to increase the sensitivity through electrode modification by exploiting nanotechnology. Notwithstanding this fact, our method still exhibits comparable linearity and LODs to those of previous studies exploiting LPME in combination with separation methods or electrochemical sensing.^20,26^ The detection time is short in almost all electrochemical studies like this work (1 min), yet LC needs longer separation times. In terms of extraction, our method is faster than previously reported microextraction techniques.^26,31,47^ Since all steps are carried out in-line and automatically, the total analysis time (∼30 min) with an extraction time of 10 min is also shorter compared to many research articles in which only extraction times are at least 20 min.^26,31,47^ In the LPME-ECD couplings reported so far, there has not been an in-depth evaluation of the sample cleanup before and after extraction for complex matrices and on the potentially deleterious effects of organic solvents on the ECD signals.^26,31^ Previous EME papers on microfluidics lacked full automation because of manual impregnation of the membranes and manual activation of some apparatus while using lengthy protocols for retrieval of the acceptor phase for detection.^10,48−50^ On the contrary, the SI−μ-EME–ECD method is entirely unattended and requires only a small volume of samples with comparable extraction recoveries (with a total analysis time of 30 min) to those of previous articles incorporating microextraction approaches.

## Conclusions

In this paper, a millifluidic SI-based device for the automation of μ-EME as a cleanup method was developed as a “front end” to in-line ECD for the first time. Utilizing the features of flow analysis, the nonsupported organic phase could be regenerated in every single measurement and all the analytical procedural steps including (i) sample loading and handling, (ii) μ-EME formation and performance, (iii) in-line pH adjustments, (iv) retrieval of the analyte-laden acceptor phase, (v) in-line injection toward the electrochemical flow cell, (vi) ECD analysis, and (vii) removal of sample and wetting film remnants were carried out fully unsupervised. In addition, analyte/sample carry-over issues and loss of membrane capacity described in previous semi-automatic EME procedures could be circumvented in this configuration. The SI−μ-EME–ECD method efficiently eliminated matrix effects and selectivity issues in the electrochemical analysis of real samples like urine. The proof-of-concept application of our computer-controlled flow system was demonstrated by the unattended cleanup, extraction, and detection of diclofenac in urine samples. Despite previous attempts to integrate EME with *in situ* ECD, the major advantage of the SI setup relies upon the minimal requirement of all operational solutions that are handled without user manipulation. Current work is underway in our research group to extend the coupling of alternative microscale extraction approaches with ECD to a broad range of analytes and troublesome matrices.

## Supporting Information Available

Detailed description of (i) reagents and samples, (ii) tubing materials, (iii) flow-setup components (via close-up) and ECD readouts, (iv) μ-EME parameters evaluated, (v) automatic operational procedure, and (vi) analytical performance of previous methods as compared with this work (PDF)

## References

[ref1] Poole C.; Mester Z.; Miró M.; Pedersen-Bjergaard S.; Pawliszyn J. Extraction for analytical scale sample preparation (IUPAC Technical Report). Pure Appl. Chem. 2016, 88, 649–687. 10.1515/pac-2015-0705.

[ref2] Alahmad W.; Sahragard A.; Varanusupakul P. Online and offline preconcentration techniques on paper-based analytical devices for ultrasensitive chemical and biochemical analysis: A review. Biosens. Bioelectron. 2021, 194, 113574 10.1016/j.bios.2021.113574. 34474275

[ref3] Drouin N.; Kubáň P.; Rudaz S.; Pedersen-Bjergaard S.; Schappler J. Electromembrane extraction: Overview of the last decade. TrAC, Trends Anal. Chem. 2019, 113, 357–363. 10.1016/j.trac.2018.10.024.

[ref4] Huang C.; Chen Z.; Gjelstad A.; Pedersen-Bjergaard S.; Shen X. Electromembrane extraction. TrAC, Trends Anal. Chem. 2017, 95, 47–56. 10.1016/j.trac.2017.07.027.

[ref5] Lee J.; Lee H. K.; Rasmussen K. E.; Pedersen-Bjergaard S. Environmental and bioanalytical applications of hollow fiber membrane liquid-phase microextraction: A review. Anal. Chim. Acta 2008, 624, 253–268. 10.1016/j.aca.2008.06.050. 18706332

[ref6] Grau J.; Azorín C.; Benedé J. L.; Chisvert A.; Salvador A. Use of green alternative solvents in dispersive liquid-liquid microextraction: A review. J. Sep. Sci. 2022, 45, 210–222. 10.1002/jssc.202100609. 34490730

[ref7] Shang Q.; Mei H.; Huang C.; Shen X. Fundamentals, operations and applications of electromembrane extraction: An overview of reviews. Microchem. J. 2022, 181, 107751 10.1016/j.microc.2022.107751.

[ref8] Wan L.; Lin B.; Zhu R.; Huang C.; Pedersen-Bjergaard S.; Shen X. Liquid-phase microextraction or electromembrane extraction?. Anal. Chem. 2019, 91, 8267–8273. 10.1021/acs.analchem.9b00946. 31141346

[ref9] Gjelstad A.; Andersen T. M.; Rasmussen K. E.; Pedersen-Bjergaard S. Microextraction across supported liquid membranes forced by pH gradients and electrical fields. J. Chromatogr. A 2007, 1157, 38–45. 10.1016/j.chroma.2007.05.007. 17521660

[ref10] Zarghampour F.; Yamini Y.; Baharfar M.; Faraji M. Simultaneous extraction of acidic and basic drugs via on-chip electromembrane extraction using a single-compartment microfluidic device. Analyst 2019, 144, 1159–1166. 10.1039/C8AN01668B. 30539185

[ref11] Hylton K.; Mitra S. A microfluidic hollow fiber membrane extractor for arsenic(V) detection. Anal. Chim. Acta 2008, 607, 45–49. 10.1016/j.aca.2007.11.039. 18155408

[ref12] Nitiyanontakit S.; Varanusupakul P.; Miró M. Hybrid flow analyzer for automatic hollow-fiber-assisted ionic liquid-based liquid-phase microextraction with in-line membrane regeneration. Anal. Bioanal. Chem. 2013, 405, 3279–3288. 10.1007/s00216-013-6744-1. 23386000

[ref13] Larsson N.; Petersson E.; Rylander M.; Jönsson J. Å. Continuous flow hollow fiber liquid-phase microextraction and monitoring of NSAID pharmaceuticals in a sewage treatment plant effluent. Anal. Methods 2009, 1, 59–67. 10.1039/b9ay00015a. 32938143

[ref14] Petersen N. J.; Jensen H.; Hansen S. H.; Foss S. T.; Snakenborg D.; Pedersen-Bjergaard S. On-chip electro membrane extraction. Microfluid. Nanofluid. 2010, 9, 881–888. 10.1007/s10404-010-0603-6. 21142026

[ref15] Ramos-Payán M.; Murillo E. S.; Coello J.; Bello-López M. A. A comprehensive study of a new versatile microchip device based liquid phase microextraction for stopped-flow and double-flow conditions. J. Chromatogr. A 2018, 1556, 29–36. 10.1016/j.chroma.2018.04.051. 29729862

[ref16] Hansen E. H.; Miró M. How flow-injection analysis (FIA) over the past 25 years has changed our way of performing chemical analyses. TrAC, Trends Anal. Chem. 2007, 26, 18–26. 10.1016/j.trac.2006.07.010.

[ref17] Miró M.; Hansen E. H. Recent advances and future prospects of mesofluidic Lab-on-a-Valve platforms in analytical sciences–A critical review. Anal. Chim. Acta 2012, 750, 3–15. 10.1016/j.aca.2012.03.049. 23062425

[ref18] Horstkotte B.; Miró M.; Solich P. Where are modern flow techniques heading to?. Anal. Bioanal. Chem. 2018, 410, 6361–6370. 10.1007/s00216-018-1285-2. 30083907

[ref19] Worawit C.; Cocovi-Solberg D. J.; Varanusupakul P.; Miró M. In-line carbon nanofiber reinforced hollow fiber-mediated liquid phase microextraction using a 3D printed extraction platform as a front end to liquid chromatography for automatic sample preparation and analysis: a proof of concept study. Talanta 2018, 185, 611–619. 10.1016/j.talanta.2018.04.007. 29759249

[ref20] Carrasco-Correa E. J.; Kubáň P.; Cocovi-Solberg D. J.; Miró M. Fully automated electric-field-driven liquid phase microextraction system with renewable organic membrane as a front end to high performance liquid chromatography. Anal. Chem. 2019, 91, 10808–10815. 10.1021/acs.analchem.9b02453. 31307195

[ref21] Fernández E.; Vidal L.; Martín-Yerga D.; Blanco M. D. C.; Canals A.; Costa-García A. Screen-printed electrode based electrochemical detector coupled with ionic liquid dispersive liquid–liquid microextraction and microvolume back-extraction for determination of mercury in water samples. Talanta 2015, 135, 34–40. 10.1016/j.talanta.2014.11.069. 25640123

[ref22] Ahmar H.; Shahvandi S. K. Determination of 4-Nitrobenzaldehyde in Water Samples by Combination of Switchable Solvent Based Microextraction and Electrochemical Detection on MWCNTs Modified Electrode. Electroanalysis 2019, 31, 1238–1244. 10.1002/elan.201800451.

[ref23] Shahraki S.; Ahmar H.; Nejati-Yazdinejad M. Electrochemical determination of nitrazepam by switchable solvent based liquid-liquid microextraction combined with differential pulse voltammetry. Microchem. J. 2018, 142, 229–235. 10.1016/j.microc.2018.07.003.

[ref24] Hrdlička V.; Barek J.; Navrátil T. Differential pulse voltammetric determination of homovanillic acid as a tumor biomarker in human urine after hollow fiber-based liquid-phase microextraction. Talanta 2021, 221, 121594 10.1016/j.talanta.2020.121594. 33076128

[ref25] Nomngongo P. N.; Ngila J. C. Hollow fiber solid phase microextraction coupled to square wave anodic stripping voltammetry for selective preconcentration and determination of trace levels of mercury in liquid fuel samples. J. Iran. Chem. Soc 2015, 12, 2141–2147. 10.1007/s13738-015-0691-z.

[ref26] Mofidi Z.; Norouzi P.; Sajadian M.; Ganjali M. R. Simultaneous extraction and determination of trace amounts of diclofenac from whole blood using supported liquid membrane microextraction and fast Fourier transform voltammetry. J. Sep. Sci. 2018, 41, 1644–1650. 29350466 10.1002/jssc.201701119

[ref27] Ahmar H.; Tabani H.; Koruni M. H.; Davarani S. S. H.; Fakhari A. R. A new platform for sensing urinary morphine based on carrier assisted electromembrane extraction followed by adsorptive stripping voltammetric detection on screen-printed electrode. Biosens. Bioelectron. 2014, 54, 189–194. 10.1016/j.bios.2013.10.035. 24280048

[ref28] Tahmasebi Z.; Davarani S. S. H.; Asgharinezhad A. A. Highly efficient electrochemical determination of propylthiouracil in urine samples after selective electromembrane extraction by copper nanoparticles-decorated hollow fibers. Biosens. Bioelectron. 2018, 114, 66–71. 10.1016/j.bios.2018.05.014. 29778003

[ref29] Norouzi P.; Akmal M. R.; Mofidi Z.; Larijani B.; Ganjali M. R.; Ebrahimi M. Low-voltage online stimulated microextraction of glibenclamide from whole blood. Microchem. J. 2019, 148, 759–766. 10.1016/j.microc.2019.05.009.

[ref30] Mofidi Z.; Norouzi P.; Larijani B.; Seidi S.; Ganjali M. R.; Morshedi M. Simultaneous determination and extraction of ultra- trace amounts of estradiol valerate from whole blood using FFT square wave voltammetry and low-voltage electrically enhanced microextraction techniques. J. Electroanal. Chem. 2018, 813, 83–91. 10.1016/j.jelechem.2018.01.048.

[ref31] Mofidi Z.; Norouzi P.; Seidi S.; Ganjali M. R. Determination of diclofenac using electromembrane extraction coupled with stripping FFT continuous cyclic voltammetry. Anal. Chim. Acta 2017, 972, 38–45. 10.1016/j.aca.2017.04.011. 28495094

[ref32] Andreasen S. Z.; Sanger K.; Jendresen C. B.; Nielsen A. T.; Emnéus J.; Boisen A.; Zor K. Extraction, enrichment, and in situ electrochemical detection on lab-on-a-disc: monitoring the production of a bacterial secondary metabolite. ACS Sens. 2019, 4, 398–405. 10.1021/acssensors.8b01277. 30525464

[ref33] Kubáň P.; Boček P. Preconcentration in micro-electromembrane extraction across free liquid membranes. Anal. Chim. Acta 2014, 848, 43–50. 10.1016/j.aca.2014.07.037. 25263115

[ref34] Kubáň P. Salt removal from microliter sample volumes by multiple phase microelectromembrane extractions across free liquid membranes. Anal. Chem. 2017, 89, 8476–8483. 10.1021/acs.analchem.7b02017. 28722395

[ref35] Cocovi-Solberg D. J.; Miró M.; et al. CocoSoft: educational software for automation in the analytical chemistry laboratory. Anal. Bioanal. Chem. 2015, 21, 6227–6233. 10.1007/s00216-015-8834-8 26143060

[ref36] Sasal A.; Tyszczuk-Rotko K.; Wójciak M.; Sowa I. First Electrochemical Sensor (Screen-Printed Carbon Electrode Modified with Carboxyl Functionalized Multiwalled Carbon Nanotubes) for Ultratrace Determination of Diclofenac. Materials 2020, 13, 781 10.3390/ma13030781. PMC7040793 32046335

[ref37] Lavanya N.; Fazio E.; Neri F.; Bonavita A.; Leonardi S. G.; Neri G.; Sekar C. Electrochemical sensor for simultaneous determination of ascorbic acid, uric acid and folic acid based on Mn-SnO_2_ nanoparticles modified glassy carbon electrode. J. Electroanal. Chem. 2016, 770, 23–32. 10.1016/j.jelechem.2016.03.017.

[ref38] Feng S.; Yu L.; Yan M.; Ye J.; Huang J.; Yang X. Holey nitrogen-doped graphene aerogel for simultaneously electrochemical determination of ascorbic acid, dopamine and uric acid. Talanta 2021, 224, 121851 10.1016/j.talanta.2020.121851. 33379067

[ref39] Chemspider database, Royal Society of Chemistry, 2022, www.chemspider.com.

[ref40] Dvořák M.; Seip K. F.; Pedersen-Bjergaard S.; Kubáň P. Semi-automated set-up for exhaustive micro-electromembrane extractions of basic drugs from biological fluids. Anal. Chim. Acta 2018, 1005, 34–42. 10.1016/j.aca.2017.11.081. 29389317

[ref41] Kubáň P.; Boček P. The effects of electrolysis on operational solutions in electromembrane extraction: the role of acceptor solution. J. Chromatogr. A 2015, 1398, 11–19. 10.1016/j.chroma.2015.04.024. 25937132

[ref42] Chuntib P.; Themsirimongkon S.; Saipanya S.; Jakmunee J. Sequential injection differential pulse voltammetric method based on screen printed carbon electrode modified with carbon nanotube/Nafion for sensitive determination of paraquat. Talanta 2017, 170, 1–8. 10.1016/j.talanta.2017.03.073. 28501144

[ref43] Davies N. M.; Anderson K. E. Clinical pharmacokinetics of diclofenac. Clin. Pharmacokinet. 1997, 33, 184–213. 10.2165/00003088-199733030-00003. 9314611

[ref44] Ensafi A. A.; Izadi M.; Karimi-Maleh H. Sensitive voltammetric determination of diclofenac using room-temperature ionic liquid-modified carbon nanotubes paste electrode. Ionics 2013, 19, 137–144. 10.1007/s11581-012-0705-0.

[ref45] Chethana B. K.; Basavanna S.; Arthoba Naik Y. Voltammetric determination of diclofenac sodium using tyrosine-modified carbon paste electrode. Ind. Eng. Chem. Res. 2012, 51, 10287–10295. 10.1021/ie202921e.

[ref46] Goodarzian M.; Khalilzade M. A.; Karimi F.; Kumar Gupta V.; Keyvanfard M.; Bagheri H.; Fouladgar M. Square wave voltammetric determination of diclofenac in liquid phase using a novel ionic liquid multiwall carbon nanotubes paste electrode. J. Mol. Liq. 2014, 197, 114–119. 10.1016/j.molliq.2014.04.037.

[ref47] Ghani M.; Ghoreishi S. M.; Salehinia S.; Mousavi N.; Ansarinejad H. Electrochemically decorated network-like cobalt oxide nanosheets on nickel oxide nanoworms substrate as a sorbent for the thin film microextraction of diclofenac. Microchem. J. 2019, 146, 149–156. 10.1016/j.microc.2018.12.044.

[ref48] Seidi S.; Ranjbar M. H.; Baharfar M.; Shanehsaz M.; Tajik M. A promising design of microfluidic electromembrane extraction coupled with sensitive colorimetric detection for colorless compounds based on quantum dots fluorescence. Talanta 2019, 194, 298–307. 10.1016/j.talanta.2018.10.046. 30609534

[ref49] Baharfar M.; Yamini Y.; Seidi S.; Arain M. B. Approach for downscaling of electromembrane extraction as a lab on-a-chip device followed by sensitive red-green-blue detection. Anal. Chem. 2018, 90, 8478–8486. 10.1021/acs.analchem.8b01224. 29847097

[ref50] Zarghampour F.; Yamini Y.; Baharfar M.; Javadian G.; Faraji M. On-chip electromembrane extraction followed by sensitive digital image-based colorimetry for determination of trace amounts of Cr (VI). Anal. Methods 2020, 12, 483–490. 10.1039/C9AY02328C.

