# Correction to Programmable Millifluidic Platform Integrating Automatic Electromembrane Extraction Cleanup and In-Line Electrochemical Detection: A Proof of Concept

**DOI:** 10.1021/acssensors.3c00786

**Published:** 2023-05-18

**Authors:** Ali Sahragard, Miloš Dvořák, Enrique J. Carrasco-Correa, Pakorn Varanusupakul, Pavel Kubáň, Manuel Miró

We regret that the fourth author’s name was misspelled in the final publication (both the main article and the Supporting Information file). It should read “Pakorn Varanusupakul” rather than “Pakorn Varanasupakul”.

## Supporting Information Available

Complete SI file with corrected author name (PDF)

