# Supplementary material for: Correction to Programmable Millifluidic Platform Integrating Automatic Electromembrane Extraction Cleanup and In-Line Electrochemical Detection: A Proof of Concept
Source: ACS Sens. 2023 May 18;8(6):2410. doi: 10.1021/acssensors.3c00786 (PMC10294248; doi:10.1021/acssensors.3c00786)
Supplement: Supplementary file 1 — se3c00786_si_001.pdf [file se3c00786_si_001.pdf]

Supporting information file

Programmable millifluidic platform integrating automatic electromembrane extraction clean-up and in-line electrochemical detection: A proof of concept

Ali Sahragard[a], Miloš Dvořák[b], Enrique J. Carrasco-Correa[c], Pakorn Varanusupakul[a], Pavel Kubáň[b] and Manuel Miró[*d]

[a]Department of Chemistry, Faculty of Science, Chulalongkorn University, Bangkok, Thailand.

[b]Institute of Analytical Chemistry of the Czech Academy of Sciences, Veveří 97, CZ-60200 Brno, Czech Republic.

[c]CLECEM group, Department of Analytical Chemistry, University of Valencia, C/Doctor Moliner 50, 46100 Burjassot Valencia, Spain

[d]FI-TRACE Group, Department of Chemistry, Faculty of Science, University of the Balearic Islands, Carretera de Valldemossa km 7.5, E-07122 Palma de Mallorca, Illes Balears, Spain.

Number of pages: 18

Number of figures: 6

Number of tables: 2

*Corresponding author. **E-mail address: manuel.miro@uib.es**

Reagents, standard solutions, and real samples. All chemicals were of analytical reagent grade and used with no further purification or treatment. Diclofenac sodium salt, sodium hydroxide, sodium chloride, hydrochloric acid, and sodium acetate trihydrate were all purchased from Merck KGaA (Darmstadt, Germany). Diclofenac stock solution of 1000 mg L^{-1} was prepared in water and stored at 4 °C pending use. Standard solutions of diclofenac were prepared daily by dissolving appropriate volumes of the stock solution in MilliQ water/urine sample. Ethanol, isopropanol, acetonitrile, and 1-octanol were obtained from Merck KGaA, and 1-nonanol, 1-decanol, and dodecanol were purchased from Fisher Scientific SL (Hampton, USA). Milli-Q water with a resistivity of >18 MΩ·cm was obtained through a Milli-Q® system (Merck-Millipore, Germany). A benchtop pH meter with a glass electrode (PC2700, EUTECH, Fisher Scientific SL) was used to control the pH of the reagent solutions and samples.

Urine samples were collected from three volunteers aged >18 years who signed a written informed consent. One individual is known to suffer from hypokalemic periodic paralysis. These volunteers took a 50 mg diclofenac sodium tablet and their urine samples were collected after 2 hours based on the short elimination half-life of this drug,[1] and stored at 4 °C. The pooled blank urine sample used in all optimization and calibration steps was obtained via a 12-h collection and stored at 4 °C. This research project was approved by the Research Ethics Committee of the Balearic Islands (ID no. IB 3776/18 PI).

Extraction Recovery (ER): The absolute extraction recovery represents the mole percentage of an analyte extracted through the proposed method and is calculated by the following equation.

$$ER\ (\%)= \frac{n_{a,final}}{n_{s,initial}} \times 100\ \%= \left(\frac{V_a}{V_s}\right) \left(\frac{C_{a,final}}{C_{s,initial}}\right) \times 100\ \%$$

where $n_{s,initial}$ and $n_{a,final}$ are the number of moles of analyte originally present in the sample and the number of moles of analyte finally collected in the acceptor solution, respectively. V_a is the volume of the acceptor solution, V_s is the volume of sample solution, $C_{a,final}$ is the final concentration of analyte in the acceptor solution as obtained by external calibration, and $C_{s,initial}$ is the initial the analyte concentration in the sample solution.

Tubing materials. Fluorinated ethylene polypropylene (FEP) tubes are normally selected for conducting μ-EME studies.[2] Because slightly different building blocks and monomers are used for the synthesis of FEP and polytetrafluoroethylene (PTFE), the inner walls produced by extrusion might differ chemically. This might affect the stability of the three phases and the magnitude of the surface free energy and the van der Waals interactions with the organic solvent. To study the effect on the μ-EME recoveries of diclofenac, μ-EME phases were formed in 2.4 mm I.D. FEP and PTFE tubing. Interestingly, ER% values obtained by PTFE (48%) were as much as twice higher than those of FEP (23%) under the same extraction conditions. In addition to the inferior recoveries with the FEP tubing, aqueous phases were frequently merged across this tubing while moving μ-EME plugs towards the electrodes. Using FEP, 1-nonanol was proven to remain attached to the walls as a wetting film so strongly that the donor phase surpassed it and mixed with the acceptor phase at the front end of the in-line configuration. Based on these observations, the SI system was exclusively built from PTFE tubing.

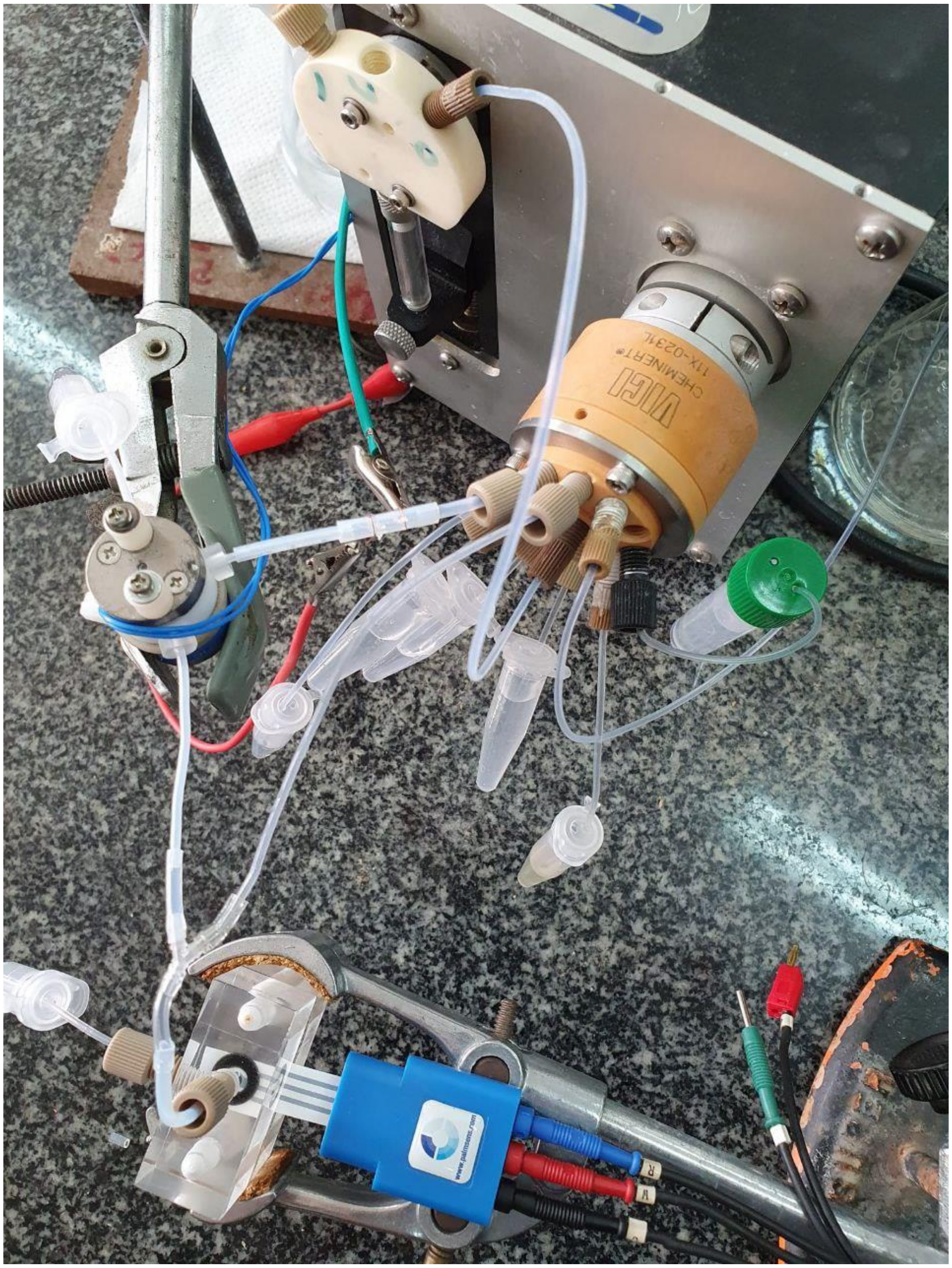

Figure S1. Close-up of the SI–μ-EME–ECD system assembled with all the components

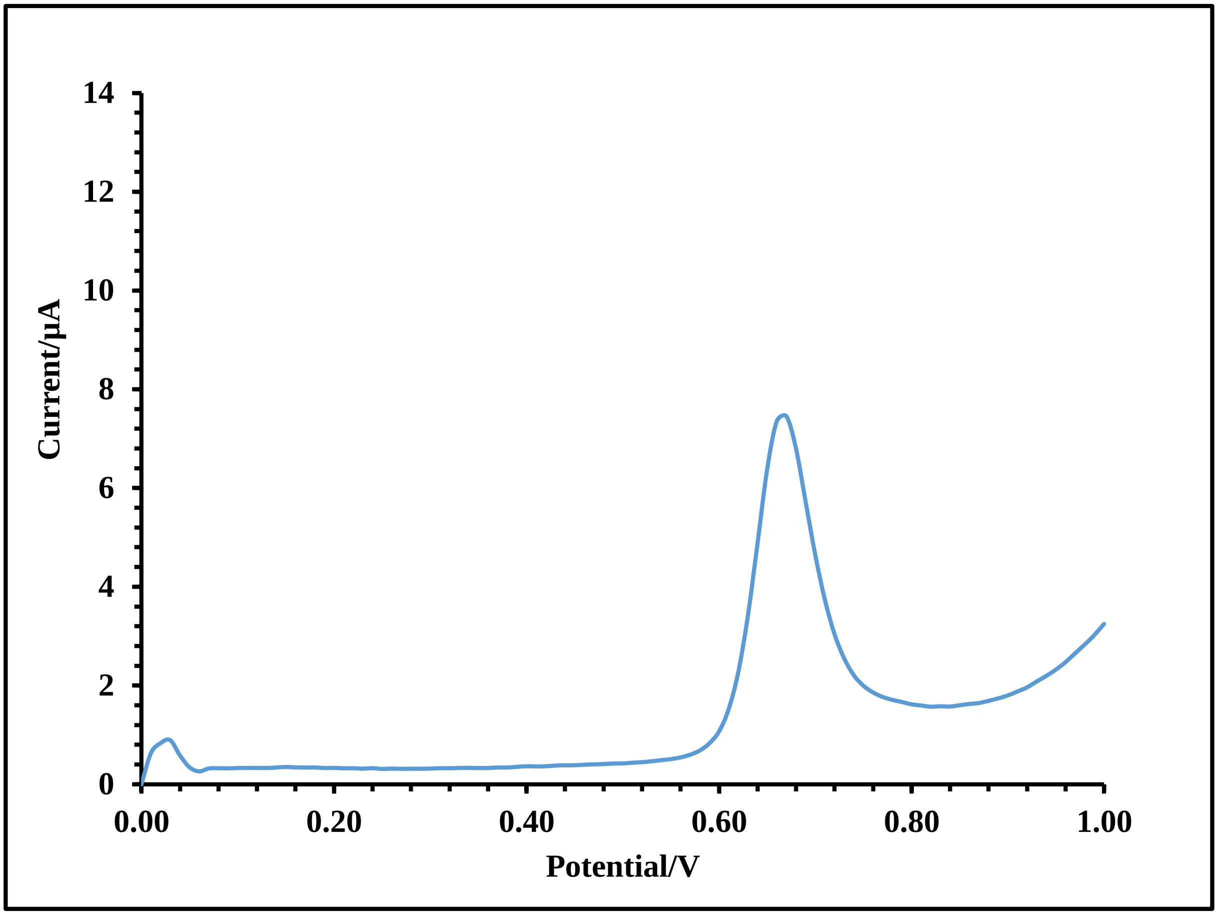

Figure S2. DPV signal of the acceptor phase (9 µL) obtained from µ-EME of 100 mM NaCl solution after mixing 1:1 with acetic acid/acetate buffer at pH = 3.75 containing 20 mg L^{-1} diclofenac.

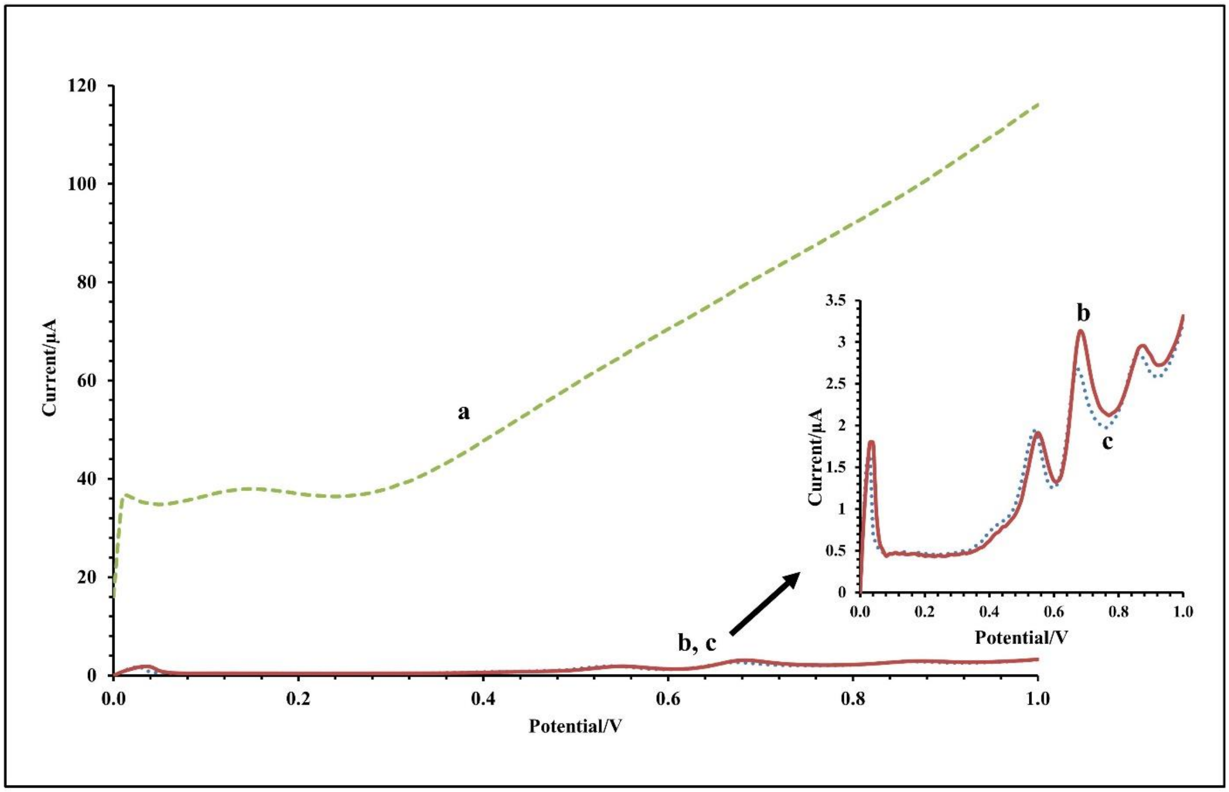

Figure S3. DPVs on SPCE after SI–µ-EME of urine spiked with 10 mg L^{-1} of diclofenac using different organic solvents in µ-EME. a) 1-octanol, b) 1-nonanol, and c) 1-decanol. Inset: magnified DPV signals for b) 1-nonanol and c) 1-decanol. Extraction conditions: donor solution, 14 µL of unprocessed urine sample containing 10 mg L^{-1} of diclofenac; organic solvent, 14 µL; acceptor solution, 14 µL of 25 mM NaOH; extraction voltage, 250 V; and extraction time, 10 min.

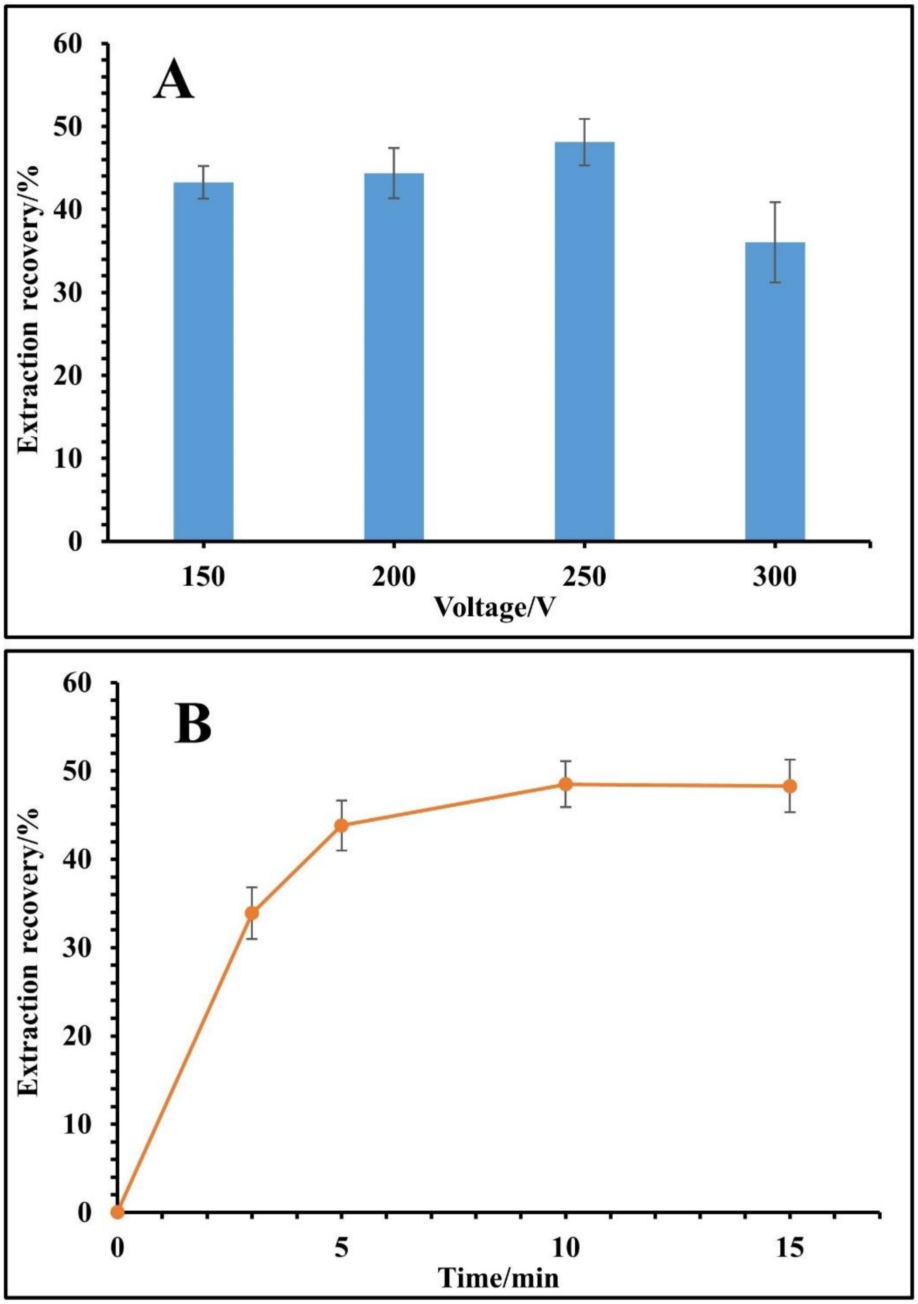

Figure S4. A) Effect of extraction voltage (150 – 300 V) on the performance of SI−μ-EME–ECD of diclofenac (*n* = *3*). Extraction conditions: acceptor solution, 14 μL of 25 mM NaOH; extraction solvent, 14 μL of 1-nonanol; donor solution, 14 μL of 10 mg L^{-1} diclofenac; extraction time, 10 min. **B**) Effect of extraction time (0 – 15 min) on the performance of SI−μ-EME–ECD of diclofenac (*n* = *3*). Extraction conditions: as for Figure S4A, extraction voltage, 250 V.

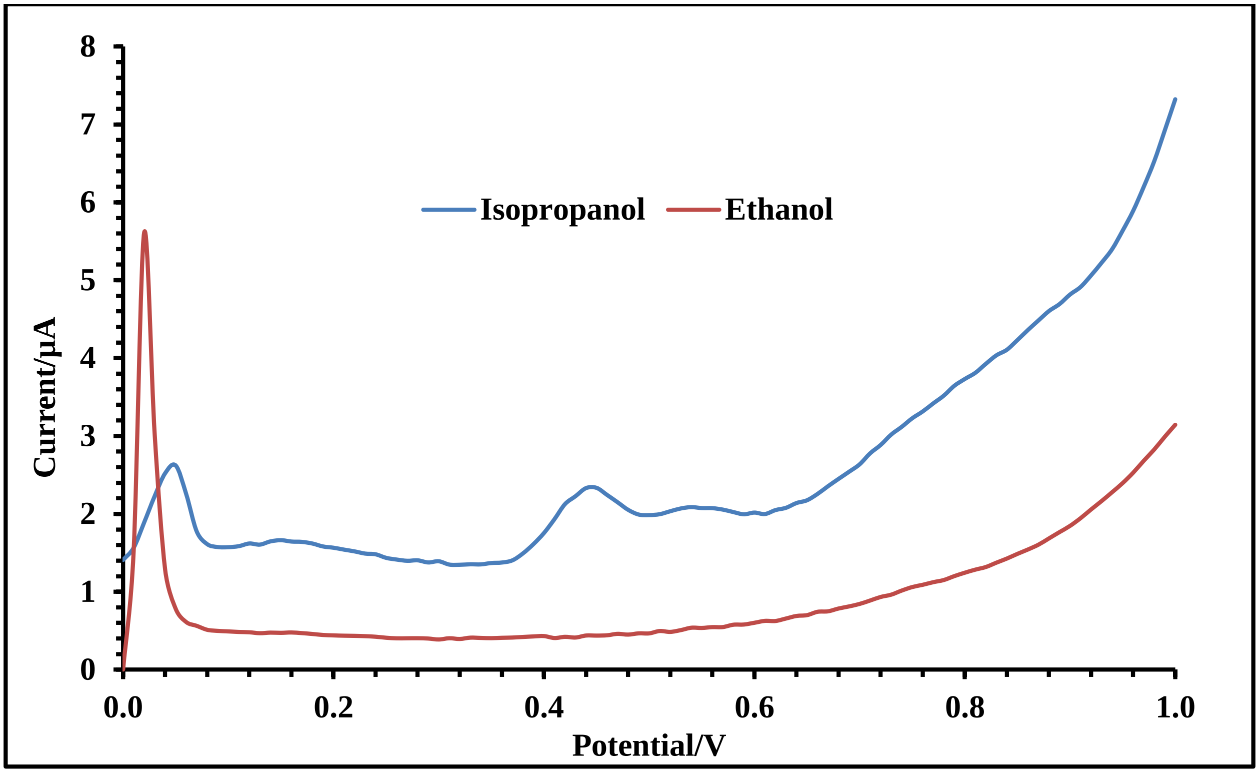

Figure S5. Comparative voltammogram of the acceptor phase (without µ-EME) after a previous µ-EME run of blank urine and washing the flow set-up with either isopropanol or ethanol.

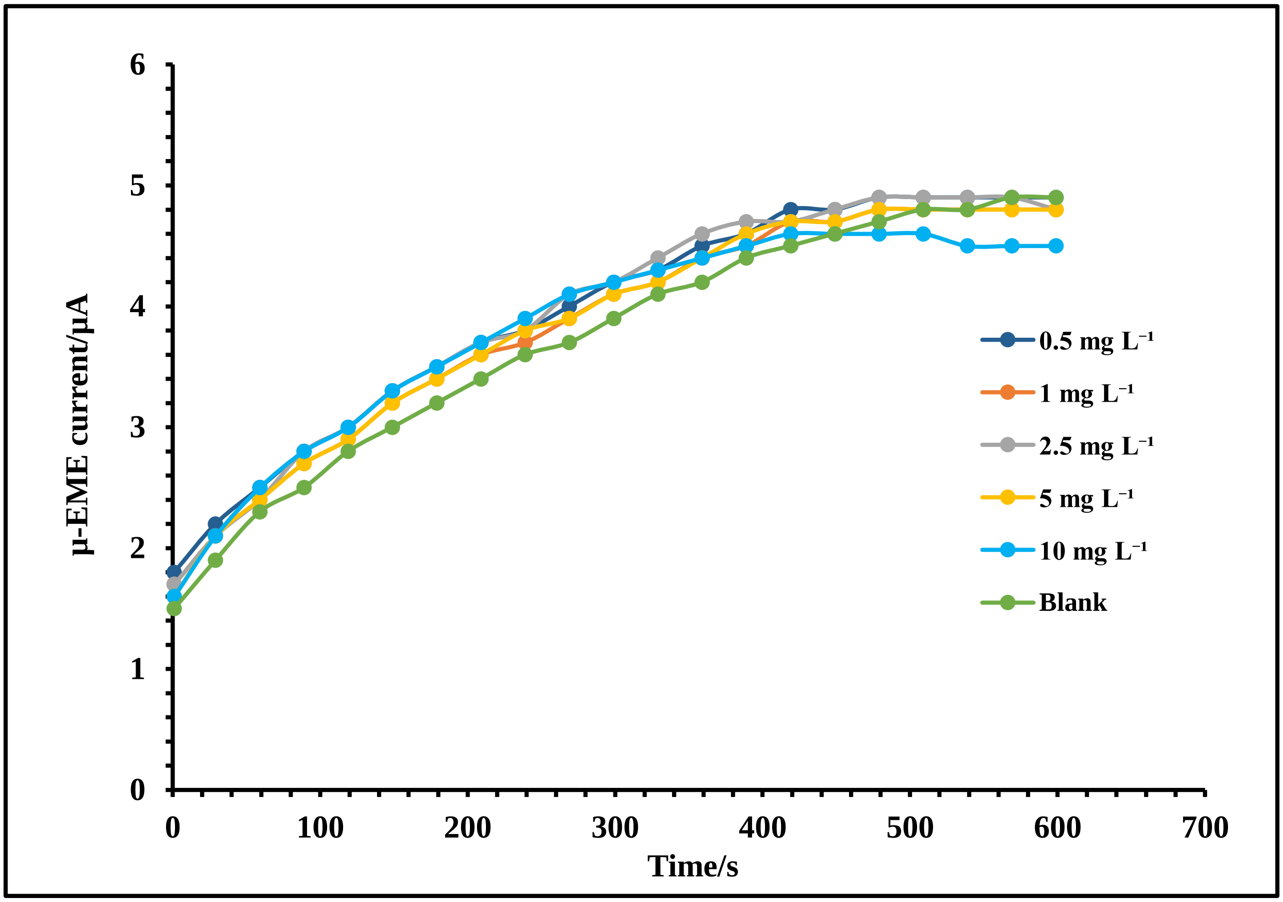

Figure S6. Recorded electric currents throughout the in-line μ-EME of increasing diclofenac concentrations (0 – 10 mg L^{-1}). Extraction conditions: donor solution, 14 μL of unprocessed urine sample containing various diclofenac concentrations; acceptor solution, 14 μL of 25 mM NaOH; extraction solvent, 14 μL of 1-nonanol; extraction voltage, 250 V; extraction time, 10 min.

Table S1. Cocosoft script for fully automatic SI−μ-EME–ECD of diclofenac in urine.

```
Loop(5)
# wash the entire system---------------------------------------------------
Fialab_uSIA_1.valve_out()
Fialab_uSIA_1.position(10)
Fialab_uSIA_1.set_speed_uL_min(2000)
Fialab_uSIA_1.dispense_uL()

Fialab_uSIA_1.output24(0)

Fialab_uSIA_1.valve_out()
Fialab_uSIA_1.position(9)
Fialab_uSIA_1.set_speed_uL_min(500)
Fialab_uSIA_1.aspirate_uL(20)

Fialab_uSIA_1.valve_out()
Fialab_uSIA_1.position(8)
Fialab_uSIA_1.set_speed_uL_min(1500)
Fialab_uSIA_1.aspirate_uL(100)

Fialab_uSIA_1.valve_out()
Fialab_uSIA_1.position(2)
Fialab_uSIA_1.set_speed_uL_min(600)
Fialab_uSIA_1.dispense_uL(100)

Loop(2)

Fialab_uSIA_1.valve_in()
Fialab_uSIA_1.set_speed_uL_min(2000)
Fialab_uSIA_1.aspirate_uL()

Fialab_uSIA_1.valve_out()
Fialab_uSIA_1.position(2)
Fialab_uSIA_1.set_speed_uL_min(500)
Fialab_uSIA_1.dispense_uL()
Loop_end()

Loop(2)
Fialab_uSIA_1.valve_out()
Fialab_uSIA_1.position(9)
Fialab_uSIA_1.set_speed_uL_min(1500)
Fialab_uSIA_1.aspirate_uL()

Fialab_uSIA_1.valve_out()
Fialab_uSIA_1.position(2)
Fialab_uSIA_1.set_speed_uL_min(500)
Fialab_uSIA_1.dispense_uL()
Loop_end()

Fialab_uSIA_1.output24(1)

Fialab_uSIA_1.valve_out()
Fialab_uSIA_1.position(9)
Fialab_uSIA_1.set_speed_uL_min(1500)
```

```
Fialab_uSIA_1.aspirate_uL(40)

Fialab_uSIA_1.valve_out()
Fialab_uSIA_1.position(8)
Fialab_uSIA_1.set_speed_uL_min(1500)
Fialab_uSIA_1.aspirate_uL(100)

Fialab_uSIA_1.valve_out()
Fialab_uSIA_1.position(2)
Fialab_uSIA_1.set_speed_uL_min(600)
Fialab_uSIA_1.dispense_uL(105)

Loop(2)
Fialab_uSIA_1.valve_in()
Fialab_uSIA_1.set_speed_uL_min(2000)
Fialab_uSIA_1.aspirate_uL()

Fialab_uSIA_1.valve_out()
Fialab_uSIA_1.position(2)
Fialab_uSIA_1.set_speed_uL_min(500)
Fialab_uSIA_1.dispense_uL()
Loop_end()

Loop(2)
Fialab_uSIA_1.valve_out()
Fialab_uSIA_1.position(9)
Fialab_uSIA_1.set_speed_uL_min(1500)
Fialab_uSIA_1.aspirate_uL()

Fialab_uSIA_1.valve_out()
Fialab_uSIA_1.position(2)
Fialab_uSIA_1.set_speed_uL_min(600)
Fialab_uSIA_1.dispense_uL()
Loop_end()

Fialab_uSIA_1.valve_out()
Fialab_uSIA_1.position(10)
Fialab_uSIA_1.set_speed_uL_min(1000)
Fialab_uSIA_1.dispense_uL()

# buffer addition to the flow cell------------------------------------------------------
Fialab_uSIA_1.initialize()

Fialab_uSIA_1.valve_in()
Fialab_uSIA_1.set_speed_uL_min(2000)
Fialab_uSIA_1.aspirate_uL(50)

# air
Fialab_uSIA_1.valve_out()
Fialab_uSIA_1.position(9)
Fialab_uSIA_1.set_speed_uL_min(1500)
Fialab_uSIA_1.aspirate_uL(40)

Fialab_uSIA_1.valve_out()
Fialab_uSIA_1.position(1)
Fialab_uSIA_1.set_speed_uL_min(500)
```

```
Fialab_uSIA_1.dispense_uL(10)

Fialab_uSIA_1.valve_out()
Fialab_uSIA_1.position(10)
Fialab_uSIA_1.set_speed_uL_min(1000)
Fialab_uSIA_1.dispense_uL(10)

# buffer
Fialab_uSIA_1.valve_out()
Fialab_uSIA_1.position(4)
Fialab_uSIA_1.set_speed_uL_min(1000)
Fialab_uSIA_1.aspirate_uL(100)

Fialab_uSIA_1.valve_out()
Fialab_uSIA_1.position(1)
Fialab_uSIA_1.set_speed_uL_min(500)
Fialab_uSIA_1.dispense_uL(95)

Fialab_uSIA_1.valve_out()
Fialab_uSIA_1.position(10)
Fialab_uSIA_1.set_speed_uL_min(1000)
Fialab_uSIA_1.dispense_uL(10)

Loop(3)
Fialab_uSIA_1.valve_out()
Fialab_uSIA_1.position(9)
Fialab_uSIA_1.set_speed_uL_min(1000)
Fialab_uSIA_1.aspirate_uL(80)

Fialab_uSIA_1.valve_out()
Fialab_uSIA_1.position(1)
Fialab_uSIA_1.set_speed_uL_min(500)
Fialab_uSIA_1.dispense_uL(80)
Loop_end()

Fialab_uSIA_1.valve_out()
Fialab_uSIA_1.position(10)
Fialab_uSIA_1.set_speed_uL_min(2000)
Fialab_uSIA_1.dispense_uL()

# EME method-----------------------------------------------------
Fialab_uSIA_1.initialize()

Fialab_uSIA_1.valve_in()
Fialab_uSIA_1.set_speed_uL_min(2000)
Fialab_uSIA_1.aspirate_uL(50)

# air
Fialab_uSIA_1.valve_out()
Fialab_uSIA_1.position(9)
Fialab_uSIA_1.set_speed_uL_min(1500)
Fialab_uSIA_1.aspirate_uL(40)

# acceptor
Fialab_uSIA_1.valve_out()
Fialab_uSIA_1.position(6)
```

```
Fialab_uSIA_1.set_speed_uL_min(500)
Fialab_uSIA_1.aspirate_uL(14)

Fialab_uSIA_1.valve_out()
Fialab_uSIA_1.position(2)
Fialab_uSIA_1.set_speed_uL_min(300)
Fialab_uSIA_1.dispense_uL(14)

# organic
Fialab_uSIA_1.valve_out()
Fialab_uSIA_1.position(5)
Fialab_uSIA_1.set_speed_uL_min(30)
Fialab_uSIA_1.aspirate_uL(14)

Fialab_uSIA_1.valve_out()
Fialab_uSIA_1.position(2)
Fialab_uSIA_1.set_speed_uL_min(30)
Fialab_uSIA_1.dispense_uL(14)

# donor
Fialab_uSIA_1.valve_out()
Fialab_uSIA_1.position(7)
Fialab_uSIA_1.set_speed_uL_min(1000)
Fialab_uSIA_1.aspirate_uL(14)

Fialab_uSIA_1.valve_out()
Fialab_uSIA_1.position(2)
Fialab_uSIA_1.set_speed_uL_min(30)
Fialab_uSIA_1.dispense_uL(14)

Fialab_uSIA_1.valve_out()
Fialab_uSIA_1.position(8)
Fialab_uSIA_1.set_speed_uL_min(1500)
Fialab_uSIA_1.aspirate_uL(50)

Fialab_uSIA_1.valve_out()
Fialab_uSIA_1.position(10)
Fialab_uSIA_1.set_speed_uL_min(500)
Fialab_uSIA_1.dispense_uL(55)


Loop(2)
Fialab_uSIA_1.valve_in()
Fialab_uSIA_1.set_speed_uL_min(2000)
Fialab_uSIA_1.aspirate_uL()

Fialab_uSIA_1.valve_out()
Fialab_uSIA_1.position(10)
Fialab_uSIA_1.set_speed_uL_min(1500)
Fialab_uSIA_1.dispense_uL()
Loop_end()

# air
Fialab_uSIA_1.valve_out()
Fialab_uSIA_1.position(9)
Fialab_uSIA_1.set_speed_uL_min(300)
```

```
Fialab_uSIA_1.aspirate_uL(45)

Fialab_uSIA_1.valve_out()
Fialab_uSIA_1.position(2)
Fialab_uSIA_1.set_speed_uL_min(30)
Fialab_uSIA_1.dispense_uL(35)

Fialab_uSIA_1.valve_out()
Fialab_uSIA_1.position(3)
Fialab_uSIA_1.set_speed_uL_min(500)
Fialab_uSIA_1.aspirate_uL(50)

Fialab_uSIA_1.valve_out()
Fialab_uSIA_1.position(2)
Fialab_uSIA_1.set_speed_uL_min(30)
Fialab_uSIA_1.dispense_uL(55)

Fialab_uSIA_1.valve_out()
Fialab_uSIA_1.position(10)
Fialab_uSIA_1.set_speed_uL_min(2000)
Fialab_uSIA_1.dispense_uL()

#Delta_elektronika_1.turn_on()
#Wait(600)
#Delta_elektronika_1.turn_off()

# injection of acceptor phase through the solenoid valve----------------------------------------------------
Fialab_uSIA_1.valve_out()
Fialab_uSIA_1.position(9)
Fialab_uSIA_1.set_speed_uL_min(500)
Fialab_uSIA_1.aspirate_uL(40)

Fialab_uSIA_1.valve_out()
Fialab_uSIA_1.position(2)
Fialab_uSIA_1.set_speed_uL_min(30)
Fialab_uSIA_1.dispense_uL(35)
# flush of the rest to the waste----------------------------------------------------

Fialab_uSIA_1.output24(0)

Fialab_uSIA_1.valve_out()
Fialab_uSIA_1.position(9)
Fialab_uSIA_1.set_speed_uL_min(500)
Fialab_uSIA_1.aspirate_uL(105)

Fialab_uSIA_1.valve_out()
Fialab_uSIA_1.position(2)
Fialab_uSIA_1.set_speed_uL_min(30)
Fialab_uSIA_1.dispense_uL(105)

# injection of acceptor to the flow cell----------------------------------------------------
Fialab_uSIA_1.output24(1)

Fialab_uSIA_1.valve_out()
Fialab_uSIA_1.position(9)
Fialab_uSIA_1.set_speed_uL_min(500)
```

```
Fialab_uSIA_1.aspirate_uL(100)

Fialab_uSIA_1.valve_out()
Fialab_uSIA_1.position(2)
Fialab_uSIA_1.set_speed_uL_min(250)
Fialab_uSIA_1.dispense_uL(100)

Fialab_uSIA_1.valve_out()
Fialab_uSIA_1.position(9)
Fialab_uSIA_1.set_speed_uL_min(500)
Fialab_uSIA_1.aspirate_uL(20)

Fialab_uSIA_1.valve_out()
Fialab_uSIA_1.position(2)
Fialab_uSIA_1.set_speed_uL_min(250)
Fialab_uSIA_1.dispense_uL(20)

# ECD detection-------------------------------------------------------
Wait (60)
Click (733, 1063)
Click (1765, 278)
Click (1765, 278)
Wait (5)
Click (733, 1063)
Loop_end ()
```

Table S2. Comparison of the analytical parameters of various methods based on chromatographic separation and electrochemical detection for the determination of diclofenac in biological samples

Extraction method	Detection method	Working electrode	Electrode modifier	Extraction time (min)	linearity ($\mu g\ L^{-1}$)	LOD ($\mu g\ L^{-1}$)	RSD%	ER %	sampl es	REF
TFME[a]	High-performance liquid chromatography-ultraviolet	-	-	30	1–200	0.15	4.3–6.6	-	Urine	[3]
μ-EME[b]	High-performance liquid chromatography-ultraviolet	-	-	10	-	1200	5.3	-	Urine	[2]
-	Differential pulse voltammetry	Gold electrode	Functionalized multiwalled carbon nanotubes - gold–platinum bimetallic nanoparticles	-	159-318130	95	-	-	Urine	[4]
-	Square wave voltammetry	Glassy carbon electrode	Multi-walled carbon nanotubes-chitosan-copper complex	-	95.4-63600	6.8	1.8-2.6	-	Urine	[5]
PTFE-LPME[c]	Fast Fourier transform stripping cyclic voltammetry	Carbon paste electrode	Reduced graphene oxide	240	1000-2500	100	5.5	32	Whole blood	[6]
PTFE-EME[d]	Stripping fast Fourier transform continuous cyclic voltammetry	Carbon paste electrode	-	20	5 -1000	1.0	-	24	Whole blood	[7]
μ-EME[b]	Differential pulse voltammetry	Screen-printed carbon electrode	-	10	500-20000	180	5.7	48	Urine	This work

[a] Thin film microextraction
[b] Micro-electromembrane extraction
[c] Polytetrafluoroethylene-liquid phase microextraction
[d] Polytetrafluoroethylene-electromembrane extraction

REFERENCES

(1) Sawchuk, R. J.; Maloney, J. A.; Cartier, L. L.; Rackley, R. J.; Chan, K. K.; Lau, H. S. Analysis of diclofenac and four of its metabolites in human urine by HPLC. *Pharm. Res.* **1995**, *12* (5), 756-762.

(2) Carrasco-Correa, E.J.; Kubáň, P.; Cocovi-Solberg, D. J.; Miró, M. Fully automated electric-field-driven liquid phase microextraction system with renewable organic membrane as a front end to high performance liquid chromatography. *Anal. Chem.* **2019**, *91* (16), 10808-10815.

(3) Ghani, M.; Ghoreishi, S. M.; Salehinia, S.; Mousavi, N.; Ansarinejad, H. Electrochemically decorated network-like cobalt oxide nanosheets on nickel oxide nanoworms substrate as a sorbent for the thin film microextraction of diclofenac. *Microchem. J.* **2019**, *146*, 149-156.

(4) Eteya, M. M.; Rounaghi, G. H.; Deiminiat, B. Fabrication of a new electrochemical sensor based on AuPt bimetallic nanoparticles decorated multi-walled carbon nanotubes for determination of diclofenac. *Microchem. J.* **2019**, *144*, 254-260.

(5) Shalauddin, M.; Akhter, S.; Bagheri, S.; Abd Karim, M. S.; Adib Kadri, N.; Basirun, W. J. Immobilized copper ions on MWCNTS-Chitosan thin film: Enhanced amperometric sensor for electrochemical determination of diclofenac sodium in aqueous solution. *Int. J. Hydrog. Energy* **2017**, *42* (31), 19951-19960.

(6) Mofidi, Z.; Norouzi, P.; Sajadian, M.; Ganjali, M. R. Simultaneous extraction and determination of trace amounts of diclofenac from whole blood using supported liquid membrane microextraction and fast Fourier transform voltammetry. *J. Sep. Sci.* **2018**, *41* (7), 1644-1650.

(7) Mofidi, Z.; Norouzi, P.; Seidi, S.; Ganjali, M. R. Determination of diclofenac using electromembrane extraction coupled with stripping FFT continuous cyclic voltammetry. *Anal. Chim. Acta* **2017**, *972*, 38-45.
